# Understanding protection from SARS-CoV-2 using metabolomics

**DOI:** 10.1038/s41598-021-93260-2

**Published:** 2021-07-05

**Authors:** Elettra Barberis, Elia Amede, Matteo Tavecchia, Emilio Marengo, Micol G. Cittone, Eleonora Rizzi, Anita R. Pedrinelli, Stelvio Tonello, Rosalba Minisini, Mario Pirisi, Marcello Manfredi, Pier Paolo Sainaghi

**Affiliations:** 1 Department of Translational Medicine, University of Piemonte Orientale, Novara, Italy; 2 Center for Translational Research on Autoimmune and Allergic Diseases, University of Piemonte Orientale, Novara, Italy; 3 Department of Sciences and Technological Innovation, University of Piemonte Orientale, Alessandria, Italy; 4 Internal and Emergency Medicine Departments, Department of Translational Medicine, University of Piemonte Orientale, Novara, Italy; 5 Azienda Ospedaliero-Universitaria “Maggiore Della Carità”, Novara, Italy

**Keywords:** Metabolomics, Predictive markers

## Abstract

The COVID-19 pandemic is still raging in most countries. Although the recent mass vaccination campaign has opened a new chapter in the battle against SARS-CoV-2, the world is still far from herd immunity. There is an urgent need to identify healthy people at high risk of contracting COVID-19, as well as supplements and nutraceuticals that can reduce the risk of infection or mitigate symptoms. In the present study, a metabolic phenotype that could protect individuals from SARS-CoV-2 infection or predispose them to developing COVID-19 was investigated. Untargeted metabolomics was performed on serum samples collected from 51 healthcare workers who were in good health at the beginning of the COVID-19 outbreak in Italy, and who were later exposed to the same risk of developing COVID-19. Half of them developed COVID-19 within three weeks of the blood collection. Our results demonstrate the presence of a specific signature associated with protection from SARS-CoV-2. Circulating monolaurin, which has well-known antiviral and antibacterial properties, was higher in protected subjects, suggesting a potential defensive role against SARS-CoV-2 infection; thus, dietary supplements could boost the immune system against this infection. In addition, our data demonstrate that people with higher levels of cholesterol are at higher risk of developing COVID-19. The present study demonstrates that metabolomics can be of great help for developing personalized medicine and for supporting public healthcare strategies. Studies with larger cohorts of subjects are necessary to confirm our findings.

## Introduction

The ongoing epidemiological emergency caused by the spreading of the SARS-CoV-2 infection requires the rethinking and reorganization of our society. Although several vaccines and treatments have been developed, the world is still far from herd immunity. The identification of healthy people at high risk of contracting COVID-19 may be a good strategy to counteract this emergency. In addition, new therapies, supplements, and nutraceuticals that can reduce the risk of SARS-CoV-2 infection or mitigate the symptoms of COVID-19 are urgently needed.

Metabolic phenotyping, the simultaneous measurement of small molecules in a biological sample, can provide a comprehensive assessment of an individual’s biochemical status. This information can be applied to personalized medicine and the development of public healthcare strategies^1^.

Most COVID-19 studies have focused on the identification of new potential biomarkers to predict the outcome of the disease or to understand the mechanisms involved in the disease^2–6^. Recent research has shown that some biomolecules, such as proteins or small molecules, might be able to predict severe COVID-19. Julkunen et al. used decade-old blood samples from the UK Biobank to develop a multi-biomarker score, measured via high-throughput metabolomics, which is indicative of the risk of severe COVID-19. The study’s limitations include the ten-year conservation of the blood samples and the fact that the sample cannot be representative of the recent patient health state^7^.

The use of metabolomics analysis to assess nutritional status is promising^8–10^. In fact, foods and food ingredients play an important role in achieving or maintaining a state of wellbeing. However, the potential contributions of foods and bioactive substances to prevent COVID-19 have only been partially explored^11^. It is well known that a healthy diet can boost one’s immune system and combat this disease, and the prophylactic and therapeutic potential of supplements, vitamins, and micronutrients have already been proposed^12,13^, but no research has demonstrated the role of food substituent in fighting it^14^. The changing of the host metabolic state from a carbohydrate-dependent glycolytic state to a fat-dependent ketogenic state has been suggested as a potential prophylactic strategy and adjuvant therapy to combat SARS-CoV-2 infection^15^. Moreover, a recent study performed on a cohort of 327,720 UK participants found that the use of probiotics, omega-3 fatty acids, multivitamins, or vitamin D was associated with a lower risk of SARS-CoV-2 infection^16^.

In the present study, for the first time, a metabolic phenotype that may protect individuals from SARS-CoV-2 infection or predispose them to developing COVID-19 was investigated. Untargeted metabolomics was performed on serum samples collected from healthcare workers who were in good health at the beginning of the COVID-19 outbreak in Italy and who were later exposed to the same risk of developing COVID-19. Of these subjects, half developed COVID-19 within three weeks of the blood collection. Since the metabolic profile represents a snapshot of the biochemical status of these individuals before contracting COVID-19, there is great potential to use this information to tailor interventional strategies to prevent the infection and to identify molecules associated with high risk of developing COVID-19.

## Results

**Figure 1:**
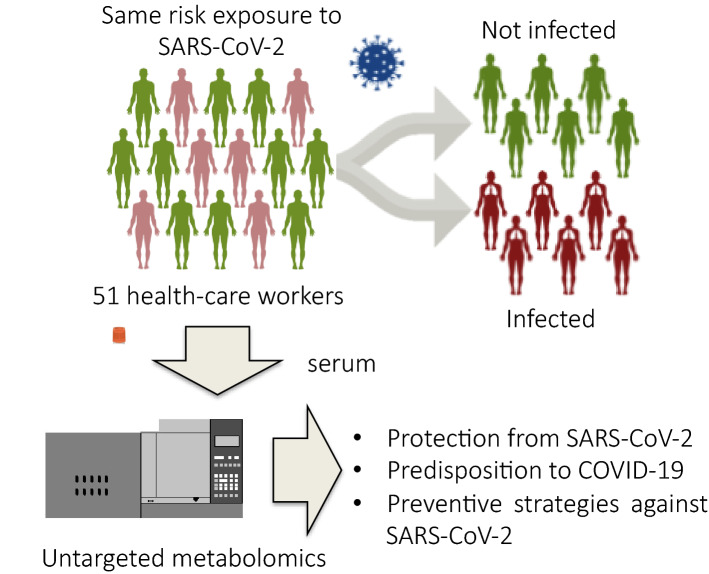
Experimental design of the study. Untargeted metabolomics was performed on 51 healthcare workers exposed to the same risk of contracting COVID-19. Twenty-four subjects developed COVID-19 in the weeks following the blood collection, while 27 subjects were more protected and did not develop the disease. The abundance of serum small molecules was used to identify the molecules that may protect individuals from SARS-CoV-2 infection or predispose them to developing it.

The aim of this research was to investigate and understand the factors and molecules that could protect individuals from SARS-CoV-2 infection or predispose them to developing COVID-19 using a metabolomics approach. Untargeted metabolomics was performed using a bi-dimensional gas chromatography/mass spectrometer (GCxGC-MS) on serum from 51 healthcare workers exposed to the same risk of contracting COVID-19. At the time of the blood collection, all the subjects were negative for SARS-CoV-2, which was determined via a nasopharyngeal swab test. Of these 51 subjects, 24 developed the COVID-19 disease in the following three weeks after the blood collection. Figure 1 provides an overview of the study. All the healthcare personnel involved in the research were working in the same department of the Novara University Hospital, in Northern Italy, the first European epicenter of the pandemic.

### Serum metabolomics signature associated with protection from SARS-CoV-2

**Figure 2:**
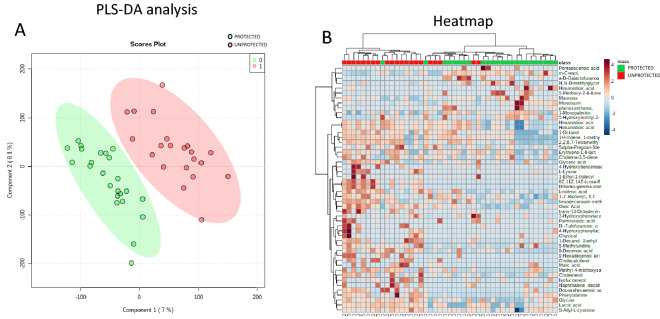
Quantified small molecules in serum from healthcare workers. Partial least square analysis (PLS-DA) performed on all the samples showed two well-defined clusters of subjects (protected = green; unprotected = red) based on the metabolomics profile (**A**). Hierarchical heat maps of quantified small molecules highlighting the two groups of samples, with subjects that contracted COVID-19 in red and subjects that were protected from SARS-CoV-2 in green (**B**).

The metabolomics analysis allowed the identification and quantification of 322 small molecules. The identification was performed not only by matching the mass signals with the mass spectra libraries, but also by manually inspecting the retention index. Serum metabolomics profiles were analyzed using multivariate analysis to identify the presence of a metabolomics signature associated with protection from SARS-CoV-2. The samples were grouped using partial least square discriminant analysis (PLS-DA). The PLS-DA provided a clear separation of the samples in terms of protection from the infection (Fig. 2A). The metabolite abundances were also analyzed and represented using a hierarchical heat map (Fig. 2B). The analysis clearly showed the presence of two groups of samples: the subjects that contracted COVID-19 in red and the subjects that were protected from SARS-CoV-2 in green.

### Biomarkers involved in protection from or predisposition to COVID-19

Univariate analysis was performed using MetaboAnalyst software to identify potential biomarkers of protection from or predisposition to the disease. A total of 41 small molecules were modulated in the two groups of samples (*p value* < 0.05, *fold change* (FC) > 1.3). The complete list of modulated molecules is reported in Supplementary Table 1. Among the up-regulated molecules in the protected subjects there were monolaurin (FC = 2.4), N,N-Dimethylglycine (FC = 2.48), 1-monopalmitin (FC = 1.58), 5-hydroxymethyl-2-furoic acid (FC = 3.54), and mannose (FC = 1.60), while dihomo-gamma-linolenic acid (FC = 0.013), L-lysine (FC = 0.059), isofucosterol (FC = 0.019), phenylalanine (FC = 0.45), oleic acid (0.48), cholesterol (FC = 0.61), lactic acid (FC = 0.60), and linolenic acid (FC = 0.58) were up-regulated in the subjects that developed the disease.

**Figure 3:**
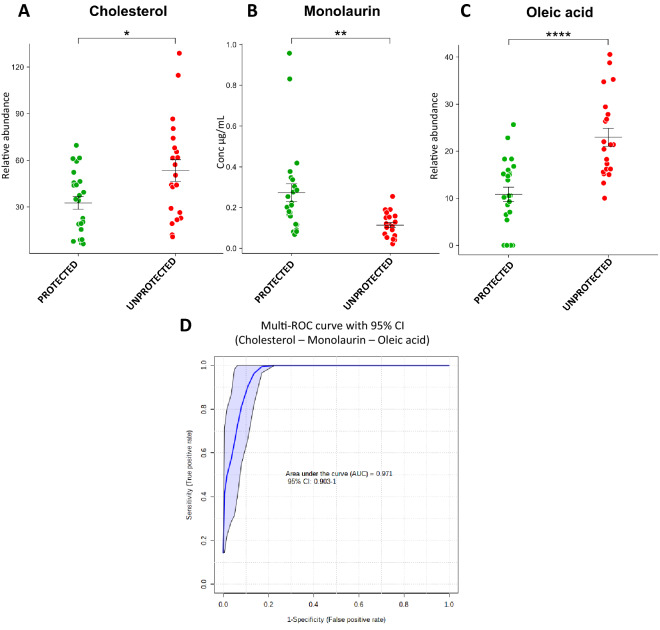
Molecules potentially involved in protecting individuals from SARS-CoV-2 infection or from COVID-19 development. Box-plot of monolaurin (**A**), cholesterol (**B**), and oleic acid (**C**) in protected and unprotected subjects. Combined ROC (**D**) of the three molecules, indicating an AUC of 0.971.

Interestingly, the analysis showed the potential protective role of some molecules, such as monolaurin and N,N-dimethylglycine, which are present at higher levels in the subjects that did not develop the disease. Monolaurin and N,N-dimethylglycine are respectively a lipid and a tertiary amino acid, that could be used as nutritional supplements. In addition, higher levels of cholesterol were identified in subjects that counteracted the infection. Moreover, the combined receiving operating curve (ROC) of monolaurin, cholesterol, and oleic acid, which were selected based on biological and statistical significance, reported an area under the curve (UAC) of 0.971, showing that these three markers might be able to predict protection from the disease (Fig. 3). In addition, statistical analysis was performed on subjects separated for gender. The results showed that cholesterol, monolaurin and oleic acid are significantly modulated between protected and unprotected subjects (Supplementary information).

Monolaurin was also determined using absolute quantification: the analysis reported an average concentration of 0.28 ± 0.24 ug/mL in the protected subjects, while the concentration was lower (0.12 ± 0.06 ug/mL) in the personnel that developed the disease after a few weeks, including asymptomatic subjects.

We next investigated the possibility of identifying subjects predisposed to COVID-19 based on the molecular signatures of small molecules. We built a random forest machine learning model based on metabolomic data from 16 protected and 16 predisposed subjects that were randomly selected from our cohort. We then tested the model on the remaining 13 subjects, reaching an average area under the curve of 0.96 (20 iterations).

### Fatty acids and steroids are potentially associated with the protection from and predisposition to COVID-19

**Figure 4:**
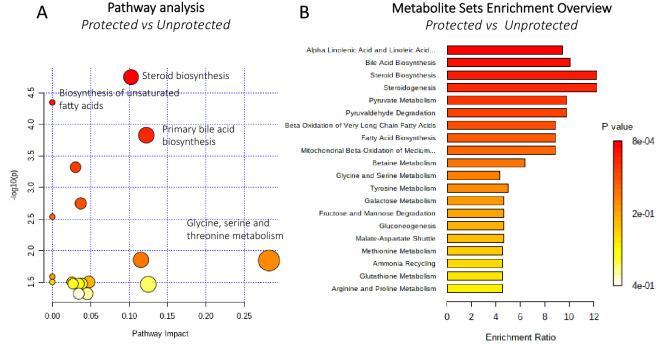
Pathways potentially involved in the protection from SARS-CoV-2. Metabolic pathway analysis performed on modulated metabolites (**A**) and metabolite sets enrichment (**B**). Steroid biosynthesis and biosynthesis of unsaturated fatty acids and amino acids may be involved in protection from COVID-19.

The pathways potentially involved in protecting individuals from SARS-CoV-2 infection were also investigated using metabolic pathways and enrichment analysis based on MetaboAnalyst 4.0 (Fig. 4). Steroid biosynthesis (cholesterol and isofucosterol), biosynthesis of unsaturated fatty acids (dihomo-gamma-linolenic acid, docosahexaenoic acid, oleic acid, linolenic acid, and monolaurin), and amino acids (glyceric acid, glycine, and N,N-Dimethylglycine) were involved in the protection from COVID-19. The metabolite set enrichment analysis shown in Fig. 4B, indicates that alpha linolenic acid and linoleic acid pathways and steroid biosynthesis were the metabolite concentrated set most altered between the two groups, confirming that fatty acids and steroids might be key factors in protecting individuals from SARS-CoV-2 infection or predisposing them to COVID-19. Table 1 reports the main pathways and the associated molecules that were modulated.

**Table 1:** Main pathways and molecules that may be involved in protection from SARS-CoV-2 infection. Fold changes and *p* values are reported.

Pathway	Molecules	Fold change	*p* value
Biosynthesis of fatty acids and unsaturated fatty acids	Dihomo-gamma-linolenic acid	0.013	1.80E−05
	Docosahexaenoic acid	0.34	3.30E−02
	Oleic acid	0.48	9.60E−06
	Linolenic acid	0.58	3.20E−02
	Monolaurin	2.4	2.00E−03
Fructose and mannose metabolism	D-Mannose	1.6	3.00E−02
Glycine, serine and threonine metabolism	Glyceric acid	0.55	4.70E−02
	Glycine	0.66	3.20E−02
	N,N-Dimethylglycine	2.48	1.00E−02
Pyruvate metabolism	Lactic acid	0.6	1.10E−03
Steroid biosynthesis	Cholesterol	0.6	1.00E−02
	Isofucosterol	0.19	4.80E−03
Tyrosine metabolism	4-Hydroxybenzeneacetic acid	0.054	2.40E−02

## Discussion

Identifying factors and molecules that might protect individuals from SARS-CoV-2 infection or predispose them to COVID-19 is still an unexplored challenge. To this aim, we investigated the metabolic phenotyping of 51 serum samples from healthcare workers. All the subjects involved in the study worked in the same department of internal medicine of the Novara University Hospital in March 2020, during the outbreak of the pandemic in Italy, and were exposed to the same risk of contracting COVID-19. At the time of the blood collection, all the subjects were negative for SARS-CoV-2, and 24 developed COVID-19 within the following three weeks. Thanks to untargeted metabolomics, we provided a comprehensive and in-depth assessment of the individuals’ biochemical status. Multivariate and monovariate comparisons of the two groups of subjects allowed the identification of a specific metabolic signature associated with the protection from and predisposition to the disease.

Notably, among modulated molecules, monolaurin levels were twice as high in subjects protected from SARS-CoV-2 infection. Monolaurin is a monoglyceride of lauric acid and a naturally occurring fatty acid ester with antibacterial and antifungal activity^17^. In addition, several studies have shown that monolaurin possesses virucidal effects against enveloped RNA and DNA viruses^18–23^. Medium-chain saturated fatty acids are highly active against enveloped viruses such as coronaviruses^24^, causing the disintegration of the viral particles^25^. The antiviral activity of monolaurin is well known: the molecule can inactivate viruses by disintegrating the viral envelope, thus not only reducing the infectivity of RNA and DNA enveloped viruses^18^ but also inhibiting the late maturation stage in the virus replication cycle, as shown by treatment of the Junin virus with lauric acid, which possesses a similar structure to SARS-CoV-2^25,26^. Lipids are indispensable for viral replication; thus, their modulation in the host cells can be used as a strategy against coronaviruses^27^. For example, lipid abundance can be modified through dietary intake to reduce inflammation and oxidative stress^28^. Virgin coconut oil, which is mainly composed of monolaurin, was able to boost the amount of lymphocyte and CD4 in vaccinated and unvaccinated broiler chickens, acting as an immunomodulator and increasing chicken immunity^29^. Another study suggested that monolaurin plays a significant role in T cell functions and signaling by altering TCR-induced LAT, PLC-γ, and AKT cluster formation, PI3K-AKT signaling axis, and calcium influx, decreasing cytokine production^30^. A recent study reported the benefit of routine use of topical coconut oil, mainly composed of monolaurin, against prototypical neonatal pathogens in preterm infants. The authors suggested that, given its significant antimicrobial activity, it is conceivable that increased concentrations of monolaurin, both locally and systemically, may improve the total defense capacity of preterm infants^31^. Moreover, the use of formic acid and monolaurin as an alternative to antibiotics in diet when piglets are challenged with Enterotoxigenic Escherichia coli has shown the potential to be an alternative to mitigate inflammatory response^32^. Lastly, a recent trial investigated the effects of virgin coconut oil (VCO), which is rich in monolaurin, on the clinical outcomes of COVID-19 patients in Indonesia. A previous study reported no side effects of VCO treatment on healthy volunteers^33^. We can speculate that higher levels of circulating monolaurin and dietary supplements may play protective roles against SARS-CoV-2 infection.

Another source of monolaurin could be from the degradation of lysophosphatidylcholine 12:0 (LPC12:0). Increased levels of LPCs were found in COVID-19 patients, while another study reported that decreased concentration of LPC16:0 could be a useful biomarker for sepsis diagnosis and mortality prediction in intensive care unit patients^34^.

Several studies have reported that patients with cardiovascular diseases, hypertension, and obesity, are at higher risk of severe manifestations and mortality due to COVID-19^35,36^. In this context, cholesterol could play a key role. In fact, our data reported higher levels of cholesterol in patients who developed COVID-19. The essential role of this molecule in viral replication and entry has been investigated in several coronaviruses, such as SARS-CoV, murine coronavirus, porcine deltacoronavirus, and infectious bronchitis virus^37–40^. Lipid rafts, which are subdomains of the plasma membrane, have an important function in viral entry into host cells. They are enriched in cholesterol and glycosphingolipids and are crucial to the interaction between the S protein and ACE2 receptor^40,41^. The presence of cholesterol in the cell membrane and viral envelope contributes to coronavirus replication. In fact, it is involved in binding and altering the oligomeric status of the N-terminal fusion peptide of SARS-CoV, which is crucial for virus entry^42,43^. Our observation is also supported by the fact that several studies associated the use of statins with a reduced risk of mortality in COVID-19 patients^44^ and lower intensive care unit admission^45^, while their use over the 30 days prior to COVID-19 infection was associated with a lower risk of developing a severe case of COVID-19 and a faster recovery from the disease^46^. Statins are widely used as cholesterol-lowering drugs and to treat hyperlipidemia^45^. It has been demonstrated that statin therapy correlates with better outcomes in both bacterial pneumonia and influenza^47–49^. Although it is not yet clear how statins reduce risks in COVID-19 patients, a possible explanation of their mechanism of action might be linked to their key function in reducing cholesterol levels. However, it is also known that these drugs can up-regulate ACE2^50^ and may further block SARS-CoV-2 infectivity via direct binding to the main protease^51^. The involvement of cholesterol and lipid pathways during the disease is also supported by several studies^5,52,53^. Wang et al. found that cholesterol 25‐hydroxylase inhibits SARS‐CoV‐2 and other coronaviruses by depleting membrane cholesterol^54,55^ Lee et al. reported lower levels of low-density lipoprotein (LDL)-Ch and high-density lipoprotein (HDL)-Ch in COVID-19 patients^56^, while another study found a dysregulation of cholesterol biosynthesis pathway in lung biopsies from COVID-19 patients and in SARS-CoV2-infected cell lines^57^. Our observation, if further validated, might have important implications not only for the development of novel pharmacological strategies, but also for the identification of subjects at higher risk of developing COVID-19.

Another interesting molecule that is more concentrated in protected subjects is N,N-dimethylglycine (DMG), which is a tertiary amino acid and a natural component of animal and plant metabolism. Several studies have demonstrated that DMG can strengthen the immune system and help the host cell to defend against bacteria, viruses, and pathogens. It has been reported that DMG stimulates B-cells to produce a higher antibody response^58^ and increases T cell and macrophage activity^59^.

Pathway analysis reported that fatty acids and steroid biosynthesis pathways are potentially associated with the protection from and the predisposition to COVID-19. Fatty acids and lipids are strongly involved both in the development of the disease and in protection from it. In fact, while dyslipidemia has been reported as a consequence of the infection^5^, in the present study, we found that a monoglyceride may play a protective and antiviral role. The steroid biosynthesis pathway is related to the modulation of cholesterol, which plays a significant role in COVID-19.

By exploring biomarker associations with severe pneumonia risk Julkunen et al.^7^ found that an increased plasma concentrations of cholesterol, omega-3 and omega-6 fatty acid levels, histidine, branched chain amino acids and albumin were associated with lower risk for contracting severe pneumonia. Although this is in contrast to our data, their samples cannot be representative of the recent patient health state.

Although, to date, a similar study is not present in literature, our research presents some limitations: the number of subjects involved is limited, the subjects were not tested for asymptomatic infection through the study period and the same individuals were not resampled to better account for intra-individual variability. However, in March 2020 the Novara Hospital was one of the first pandemic epicenters in the world, the screening procedures on asymptomatic health care professionals were not available and the emergency situation did not allow us to collect more samples to assess the intra-individual variability.

In conclusion, for the first time, we reported the presence of a metabolic phenotype specifically associated with protection from SARS-CoV-2 infection and predisposition to COVID-19. It therefore seems plausible that higher levels of circulating monolaurin, which has antiviral and antibacterial properties, may play a protective role against SARS-CoV-2 infection and that monolaurin dietary supplements could boost individuals’ immune systems and, in turn, potentially prevent infection. A randomized controlled trial of monolaurin supplements is required to confirm these observational findings before any therapeutic recommendations can be made. In addition, our data suggest that people with higher levels of cholesterol are at higher risk of developing COVID-19. Moreover, studies with larger cohorts of subjects are needed to confirm our findings. The present work demonstrated that metabolomics could be of great help to developing personalized medicine and to implementing public healthcare strategies.

## Materials and methods

### Patients

We performed a prospective observational study with collection of blood samples and clinical data about COVID-19 infections from healthcare professionals on duty during the COVID-19 emergency. All subjects underwent a blood draw between 10 and 25 March 2020 at the beginning of the first wave of the COVID-19 emergency in Italy. The enrolled subjects were reevaluated at 12 weeks for eventual COVID-19 infection during the observation period. The Institutional Review Board (Comitato Etico Interaziendale Novara) approved this study (n. RQ06320/25 March 2020) and all methods were performed in accordance with the relevant guidelines and regulations. Informed consent was obtained from all participants and/or their legal guardians and the research have been performed in accordance with the Declaration of Helsinki.

All subjects declared no signs or symptoms or any documentation of SARS-Cov-2 infection at enrollment. All the subjects were physicians or nurses on duty at the internal medicine department of Novara University Hospital and were exposed to the same risk of SARS-CoV-2 infection since the Internal Medicine Department became a COVID ward starting on 9 March 2020. All the subjects were fully dedicated to COVID-19 patients care. Additionally, during the first 2–3 weeks of March 2020 there was an extreme shortage of personal protection equipments as FFP2 and FFP3 mask and only surgical face masks were available: for this reason the exposure risk was very high during patients care.

Since the study was performed during the very beginning of COVID-19 pandemic on March 2020, due to a very limited availability, SARS-CoV-2 RT-PCR testing was limited only to symptomatic patients at emergency department. Thus, the subjects were not tested for asymptomatic infection through the study period.

Of these 51 healthy control subjects, 24 developed COVID-19 within three weeks of enrollment, which was confirmed via reverse transcription polymerase chain reaction (RT-PCR). Clinical characteristics of the patients are reported in Table 2. All the subjects developed at worst a moderate case of COVID-19, and none required oxygen or hospital admission. All subjects enrolled were alive at 12 weeks. All the subjects included in the research were Caucasian.

**Table 2:** Characteristics of the subjects included in the study.

		Not infected (protected)	Infected (unprotected)
	Total (51)	(*n* = 27)	(*n* = 24)
**Sex (N°)**			
M	20	11	9
F	32	16	16
**Age (years)**			
Mean ± SD	37.6 ± 10.6	36.5 ± 10.1	38.9 ± 10.9
Range	26–63	26–63	26–62
**Time from serum collection to diagnosis of infection (days)**			
Mean ± SD			13.3 ± 5.1
Range			6–21
Smoking	5	3	2
BMI	23.4 ± 2.5	23.4 ± 2.4	23.3 ± 1.7
**Symptoms (N° of patients)**			
Fever			14
Cough			10
Rhinorrhea			5
Headache			11
Anosmia			12
Ageusia			11
Asthenia			10
Myalgia/Arthralgia			6
Other symptoms			13
Asymptomatic			4
Symptomatic			20

### Sample preparation for metabolomics analysis

The samples were prepared as previously reported by Barberis et al.^5^. Briefly, 1 mL of an ACN/IPA/water (3:3:2) solution (Merck, Darmstad, Germany), with tridecanoic acid (Merck, Darmstad, Germany) at 1 ppm as the internal standard, was added to 30 µL of serum. Each sample was then vortexed and centrifuged at room temperature for 15 min at 14,500× *g*. The supernatant was then dried in a speed vacuum. It was then derivatized with 20 µL of methoxamine at 80 °C for 20 min and underwent sialylation with 90 µL of BSTFA (Merck, Darmstad, Germany) at 80 °C for 20 min. Then, 10 µL of hexadecane (IS) were added, and the sample was ready for GCxGC-MS analysis.

### GCxGC/TOFMS analysis

For metabolomics analysis, a LECO Pegasus BT 4D GCxGC/TOFMS instrument (Leco Corp., St. Josef, MI, USA) equipped with a LECO dual-stage quad jet thermal modulator was used. The samples were analyzed as previously reported by Barberis et al.^5,60^. Briefly, the first dimension column was a 30 m Rxi-5Sil (Restek Corp., Bellefonte, PA, USA) MS capillary column with an internal diameter of 0.25 mm and a stationary phase film thickness of 0.25 μm, while the secondary column was a 2 m Rxi-17Sil MS (Restek Corp., Bellefonte, PA, USA) with the same diameter and thickness as the first. High-purity helium (99.9999%) was used as the carrier gas with a flow rate of 1.4 mL/min. Then, 1 μL of sample was injected at 250 °C in splitless mode. The temperature program was as follows: the initial temperature was set at 70 °C for 2 min, then increased by 6 °C/min up to 160 °C, 10 °C/min up to 240 °C, and 20 °C/min to 300 °C and then maintained at this temperature for 6 min. The secondary column was kept at + 5° C relative to the gas chromatography (GC) oven temperature of the first column. The programming rate was the same for both columns. Electron impact ionization was applied (70 eV). The ion source temperature was set at 250 °C, and the mass range was 25–550 m/z with an extraction frequency of 32 kHz. The acquisition rates were 200 spectra/s and the modulation period was 4 s for the entire run. The modulator temperature offset was set at + 15 °C relative to the secondary oven temperature, while the transfer line was set at 280 °C^5,61^.

The chromatograms were acquired in total ion current (TIC) mode. The peaks with signal-to-noise (S/N) values lower than 500.0 were rejected. ChromaTOF version 5.51 was used for the raw data processing. Mass spectral assignment was performed by matching with NIST MS Search 2.3 libraries and the FiehnLib. In addition, an in-house library of standards was used to identify the small molecules. Statistical analysis was performed with Metaboanalyst 4.0 software (www.metaboanalyst.org).

During the analysis, several quality control procedures were included. Pooled samples, prepared using all the patients’ serum, were used for system suitability tests at the beginning, middle, and end of the batch. Blanks were also included. Internal standards (tridecanoic acid and hexadecane) were spiked in each sample and used for instrument stability monitoring and/or data normalization.

The absolute quantification of monolaurin (Sigma, Milano, Italy) was performed using an external calibration curve carried out in a concentration range from 0.01 to 5 ug/mL.

### Machine learning analysis

We randomly divided the samples in two cohorts composed by 32 (training) and 13 (validation) subjects. From the training cohort we selected important metabolite features with recursive feature elimination using random forest. In the random forest analysis, a 100 hundred trees were built using R package caret (version 4.6.14) with fivefold cross validation repeated for 5 times, and this whole framework was repeated for 20 times. Those selected important features were used for the random forest analysis on the independent test cohort (13 subjects).

## Supplementary Information

Supplementary Information 1.

Supplementary Information 2.
