# Supplementary material for: Understanding protection from SARS-CoV-2 using metabolomics
Source: Sci Rep. 2021 Jul 5;11:13796. doi: 10.1038/s41598-021-93260-2 (PMC8257707; doi:10.1038/s41598-021-93260-2)
Supplement: Supplementary file 1 — Supplementary Information 1. [file 41598_2021_93260_MOESM1_ESM.docx]

**Understanding protection from SARS-CoV-2 using metabolomics**

Elettra Barberis^1,2^, Elia Amede^1,2^, Matteo Tavecchia^1,2^, Emilio Marengo^2,3^, Micol G. Cittone^4,5^, Eleonora Rizzi^4,5^, Anita R. Pedrinelli^4,5^, Stelvio Tonello^1^, Rosalba Minisini^1^, Mario Pirisi^1,4,5^, Marcello Manfredi^1,2^* and Pier Paolo Sainaghi^1,4,5^

^1^ Department of Translational Medicine, University of Piemonte Orientale, Novara, Italy.

^2^ Center for Translational Research on Autoimmune and Allergic Diseases, University of Piemonte Orientale, Novara, Italy.

^3^ Department of Sciences and Technological Innovation, University of Piemonte Orientale, Alessandria, Italy.

^4^ Internal and Emergency Medicine Departments, Department of Translational Medicine, University of Piemonte Orientale, Novara, Italy.

^5^ Azienda Ospedaliero-Universitaria "Maggiore della Carità", Novara, Italy.

***** Correspondence: [marcello.manfredi@uniupo.it](mailto:marcello.manfredi@uniupo.it)

**Supplementary figure 1**: Box-plot of monolaurin for subjects separated based on gender, female (left) and male (right).

**Supplementary figure 2**: Box-plot of cholesterol for subjects separated based on gender, female (left) and male (right).

**Supplementary figure 3**: Box-plot of oleic acid for subjects separated based on gender, female (left) and male (right).
